# The Amazon River plume, a barrier to animal dispersal in the Western Tropical Atlantic

**DOI:** 10.1038/s41598-021-04165-z

**Published:** 2022-01-11

**Authors:** Everton Giachini Tosetto, Arnaud Bertrand, Sigrid Neumann-Leitão, Miodeli Nogueira Júnior

**Affiliations:** 1 Departamento de Oceanografia, Universidade Federal de Pernambuco, Recife, PE 50670-901 Brazil; 2 Institut de Recherche pour le Développement, MARBEC, Université Montpellier, CNRS, IFREMER, IRD, 34200 Sète, France; 3 Departamento de Pesca e Aquicultura, Universidade Federal Rural de Pernambuco, Recife, PE 52171-900 Brazil; 4 Departamento de Sistemática e Ecologia, Universidade Federal da Paraíba, João Pessoa, PB 58051-900 Brazil

**Keywords:** Biogeography, Biooceanography, Biodiversity

## Abstract

The dispersal of marine organisms can be restricted by a set of isolation mechanisms including hard barriers or hydrological features. In the Western Atlantic Ocean, the Amazon River discharge has been shown to act as a biogeographical barrier responsible for the differences in reef fish communities between Caribbean Sea and Northeast Brazil continental shelves. Here, we compare the diversity of all Animalia phyla from biogeographic ecoregions along the Tropical Western Atlantic continental shelf to test the hypothesis that the Amazon River plume spatially structures species diversity. For that, we used beta diversity estimators and multivariate ecological analysis on a database of species occurrence of the whole animal kingdom including 175,477 occurrences of 8,375 species from six ecoregions along the Western Tropical Atlantic. Results of the whole animal kingdom and the richest phyla showed that the Caribbean Sea and Tropical Brazil ecoregions are isolated by the Amazon River Plume, broadening and confirming the hypothesis that it acts as a soft barrier to animal dispersal in the Western Tropical Atlantic. Species sharing is larger northwestwards, in direction of the Caribbean than the opposite direction. Beyond species isolation due to local characteristics such as low salinity and high turbidity, our results suggest the dominant northwestward currents probably play a major role in animal dispersion: it enhances the flux of larvae and other planktonic organisms with reduced mobility from Brazil to Caribbean and hinders their contrary movement. Thus, the Amazon area is a strong barrier for taxa with reduced dispersal capacity, while species of pelagic taxa with active swimming may transpose it more easily.

## Introduction

Animal dispersal is typically easier in marine environments than on land because planktonic life stages, present in most marine taxa, are drifted and dispersed by currents^1^. However, a set of isolating mechanisms can restrict the dispersal of marine organisms. Hard barriers such as landmasses are the most evident, physically splitting marine habitats^2^. This was particularly observed through the isolation of the Atlantic Ocean Realm by the closure of the Tethys seaway and Isthmus of Panama from the Indo-Pacific and East Pacific oceans, respectively^2^. Soft barriers, related to hydrology and distance, may also play a significant role in limiting the movement of organisms^2^. At a global scale, large distances over the open ocean may restrict connectivity and the exchange of species almost like physical hard barriers, as observed in the isolation of the Indo-Pacific and East Pacific realms^2^, and in differences in communities from both sides of the Atlantic Ocean^3^.

At regional scales, specific physical oceanographic processes may play a major role in species distribution patterns^4^. Currents can enhance the flow of larvae and other planktonic organisms with reduced mobility. On the other hand, they may hinder dispersal against the flow^4,5^. For example, species from the Indian Ocean invaded the Atlantic realm through the Agulhas/Benguela Currents, but not the other way round^5^. Physical and chemical properties of seawater, associated with species ecological niche limitations and/or stratification of the water column, may also act as soft barrier limiting species dispersal^6,7^. The Antarctic Polar Front is an example of how temperature bounds the distribution of subtropical/temperate and polar organisms^6,7^. Freshwater discharge over the continental shelf, with associated reduced salinity and enhanced primary production and/or turbidity may also significantly change local seawater properties restricting species distribution^8,9^.

The Amazon River induces the world largest river discharge (up to 2.4 × 10^5^ m^3^ s^−1^)^10,11^. It produces a surface plume of low salinity (< 35) and high concentrations of suspended material and nutrients that spreads for thousands of kilometres over the North Brazilian Continental Shelf and adjacent open waters in the Equatorial Atlantic Ocean carried by the strong currents in the area^12,13^. In low sea-level glacial periods, the plume covered the entire water column over the continental shelf, preventing dispersal of neritic species associated with saline waters through the area^9^. In higher sea-level periods, such as the current warm period, saline water masses are present below the plume (> 30 m depth) over the continental shelf and exchange of marine organisms could occur in sub-surface, where the large Amazon reef system is present^9,14–16^. Even though the Amazon river plume (ARP) is considered the main biogeographical barrier responsible for the differences in reef fish^3,9^, shallow-water sea anemones^17^ and prosobranch gastropods^18^ communities between the Caribbean Sea and Northeast Brazil continental shelves, two western boundary systems with distinct oceanographic characteristics^19–21^. However, the divergences in species composition among these areas have never been assessed in a broader context extending to all animal phyla.

**Figure 1:**
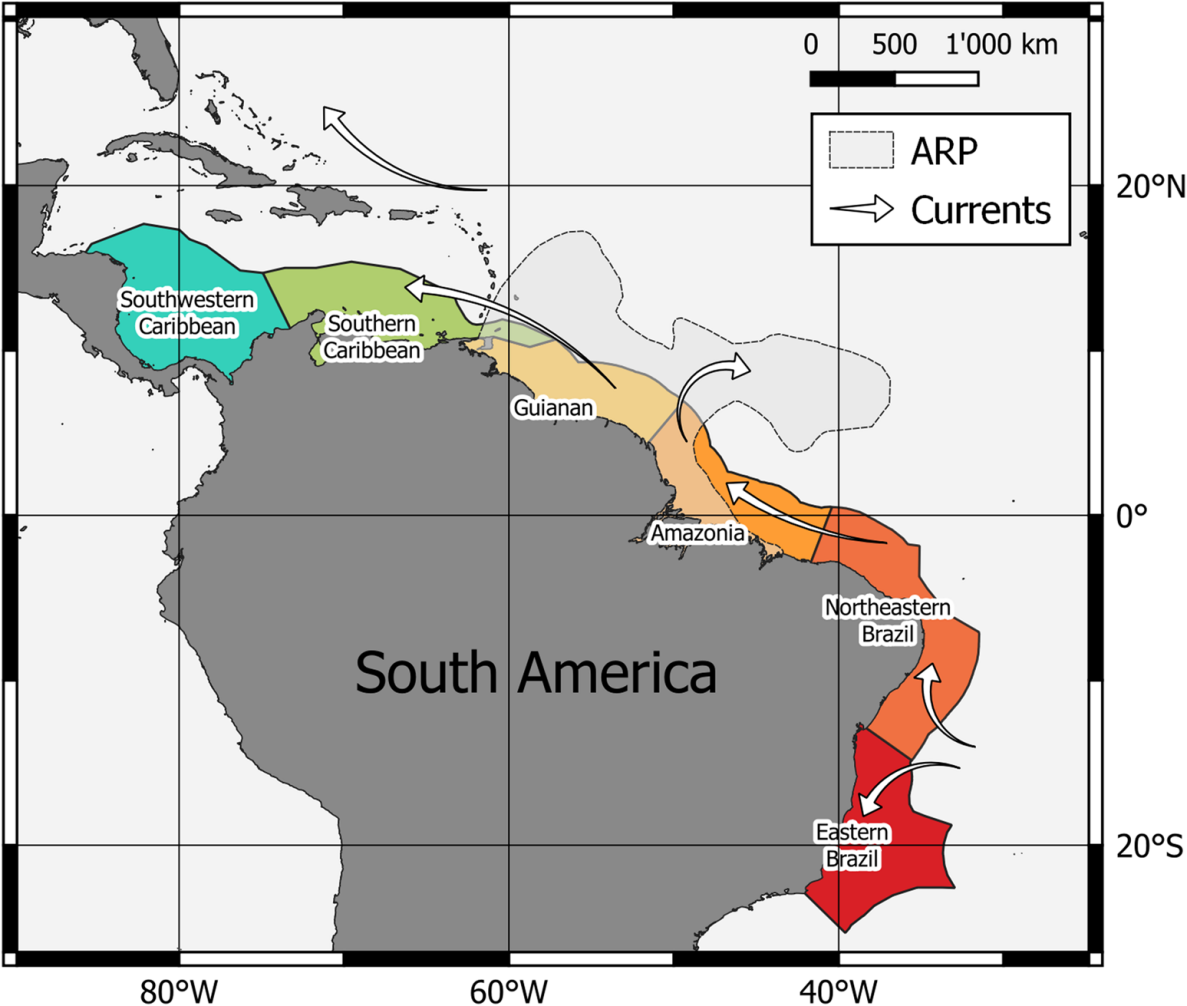
The six ecoregions from the Western Tropical Atlantic Ocean considered in the present study. Shaded area roughly represents the area influences by the ARP along the year. Arrows indicate the overall direction of surface currents. Map was developed with QGIS 3.16.5 (https://www.qgis.org).

Since the last decades, the development of large database platforms integrating small and isolated datasets of species occurrence allows exploring and producing a more comprehensive picture of the distribution and diversity of marine life at global and at regional scales^22,23^. Taking advantage of the Ocean Biogeographic Information System—OBIS^24^, an extensive database with approximately 80 million presence records of more than 156,000 marine species, here we test the hypothesis according to which, in the Tropical Western Atlantic border, the ARP spatially structures animal species diversity. For that, we compare the diversity of all Animalia phyla from biogeographic ecoregions^25^ along the Tropical Western Atlantic continental shelf (Fig. 1) analysed with geographic information system and ecological community estimators.

## Results

A total of 175,477 occurrences of 8375 animal species were recorded among the six ecoregions in the OBIS database (Table 1, 2, supplementary Table S1). The richest phylum was Arthropoda totalling 2379 species (28.4%), followed by Chordata (1986 species; 23.7%) and Mollusca (1746 species; 20.8%). Porifera, Cnidaria, Annelida and Echinodermata were also notable (ranging from 5.0 to 7.0% of total number of species), while other phyla accounted to less than 2% of the species (Table 2). Southwestern and Southern Caribbean ecoregions presented the highest species richness (4070 and 3928 species respectively; Table 2). Richness was lower in Eastern Brazil, Northeastern Brazil and Guianan (2720, 1365 and 1561 species, respectively) and the lowest value was observed in the Amazon ecoregion (756 animal species; Table 2). Among the 175,477 occurrences (Table 1, supplementary Table S1), most were recorded in Southwestern Caribbean (48.3%), Southern Caribbean (23.8%) and Eastern Brazil (11.4%). Otherwise, Amazon, Northeastern Brazil and Guianan presented 3.0, 4.2 and 9.3% of records, respectively (Table 1). Chordata was the phylum with most records (46.5%), followed by Arthropoda (19.3%), Cnidaria (13.7%) and Mollusca (11.3%). Among the 10 species with more records, eight were fish from the order Perciformes and two were soft corals from the phylum Cnidaria (supplementary Table S1).

**Table 1:** Number of occurrences in each animal phyla recorded in Ocean Biogeographic Information System (OBIS) database in February 2021.

Phylum	Southwestern Caribbean	Southern Caribbean	Guianan	Amazonia	Northeastern Brazil	Eastern Brazil	Total
Annelida	398	895	93	19	108	798	**2311**
Arthropoda	7236	14,230	4995	934	3498	2950	**33,843**
Brachiopoda	18	22	9	0	1	0	**50**
Bryozoa	5	49	1	0	5	0	**60**
Chaetognatha	16	0	0	175	84	1009	**1284**
Chordata	47,533	10,906	9039	3742	2518	7790	**81,528**
Cnidaria	15,446	5179	468	169	201	2627	**24,090**
Echinodermata	6551	2198	754	95	103	354	**10,055**
Kinorhyncha	5	3	0	0	0	0	**8**
Mollusca	7106	7033	821	130	636	4124	**19,850**
Nematoda	0	2	0	0	26	0	**28**
Nemertea	14	0	0	0	0	0	**14**
Platyhelminthes	16	1	0	0	0	0	**17**
Porifera	327	1190	42	29	203	382	**2173**
Rotifera	0	0	0	4	15	0	**19**
Sipuncula	7	79	25	1	11	0	**123**
Tardigrada	0	0	0	0	3	0	**3**
Xenacoelomorpha	21	0	0	0	0	0	**21**
**Total**	**84,699**	**41,787**	**16,247**	**5298**	**7412**	**20,034**	**175,477**

**Table 2:** Number of species in each animal phyla recorded in Ocean Biogeographic Information System (OBIS) database in February 2021.

Phylum	Southwestern Caribbean	Southern Caribbean	Guianan	Amazonia	Northeastern Brazil	Eastern Brazil	Total
Annelida	125	183	28	17	45	213	**516**
Arthropoda	859	1326	384	194	441	558	**2379**
Brachiopoda	8	4	3	0	1	0	**10**
Bryozoa	4	6	1	0	4	0	**15**
Chaetognatha	2	0	0	9	12	17	**20**
Chordata	1272	1004	711	383	434	727	**1986**
Cnidaria	343	268	88	44	93	176	**569**
Echinodermata	295	260	132	46	40	65	**446**
Kinorhyncha	5	2	0	0	0	0	**7**
Mollusca	859	707	175	42	158	834	**1746**
Nematoda	0	1	0	0	10	0	**11**
Nemertea	3	0	0	0	0	0	**3**
Platyhelminthes	10	1	0	0	0	0	**10**
Porifera	128	298	37	18	106	130	**612**
Rotifera	0	0	0	2	13	0	**14**
Sipuncula	6	10	2	1	6	0	**20**
Tardigrada	0	0	0	0	2	0	**2**
Xenacoelomorpha	9	0	0	0	0	0	**9**
**Total**	**3928**	**4070**	**1561**	**756**	**1365**	**2720**	**8375**

**Figure 2:**
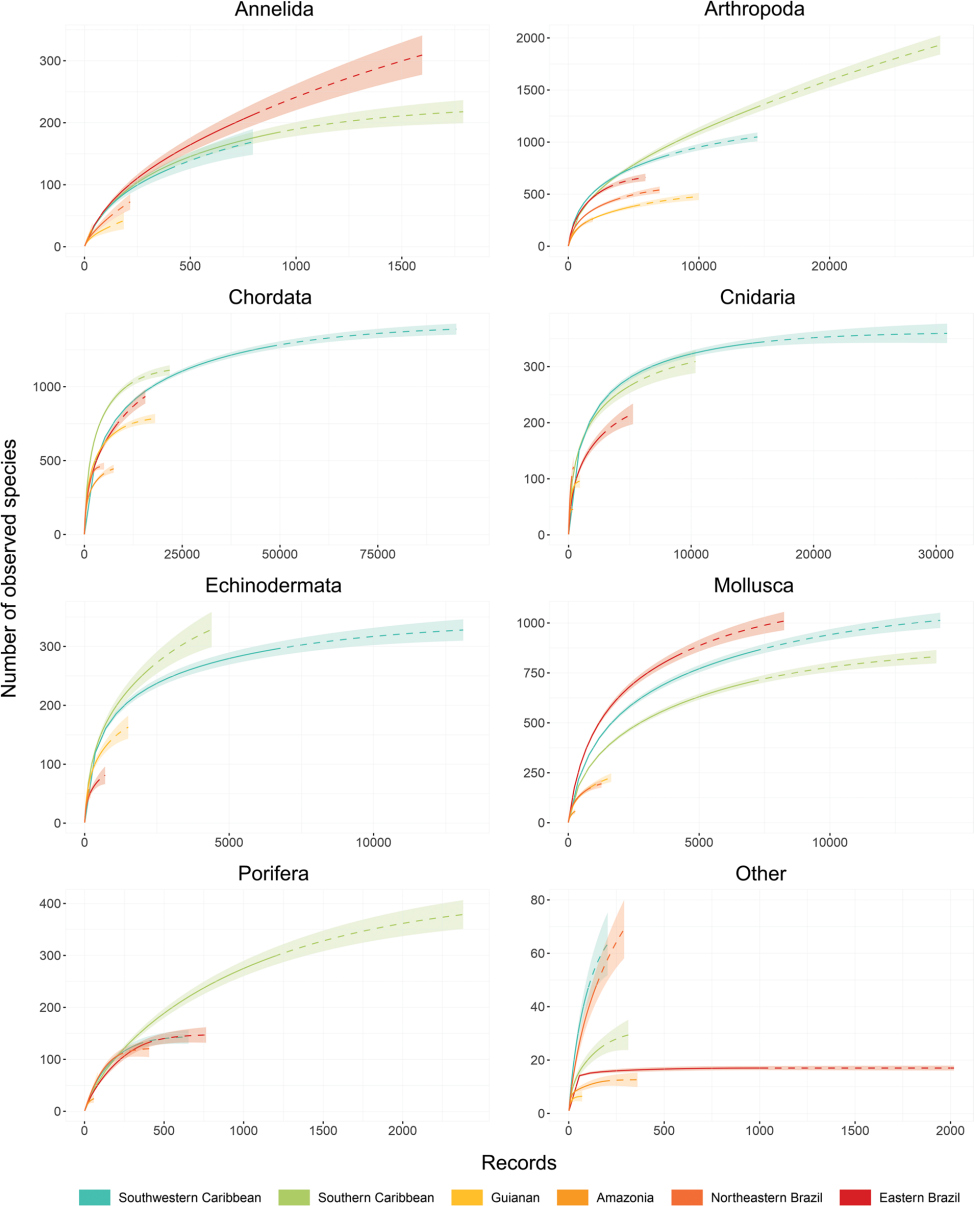
Species accumulation plots (solid lines), extrapolation (dashed lines) and 95% confidence intervals (shaded areas) for the richest phyla, and remaining ones (11 phyla), in the six ecoregions of Western Tropical Atlantic Ocean.

Species accumulation curves for the richest phyla did not reach an asymptote in any of the ecoregions, indicating that current knowledge on animal biodiversity found in the OBIS database is still incomplete for Tropical Western Atlantic (Fig. 2). Biodiversity knowledge shortfall^26^ is however smaller for Chordata in the six ecoregions, Arthropoda (except for Southern Caribbean, where the curve keeps rising despite the high number of species), Cnidaria and Mollusca in Southern and Southwestern Caribbean and Eastern Brazil, and Echinodermata in Southwestern Caribbean (Fig. 2). Actually, when extrapolating the curves to reach the asymptote, the pattern of species richness among region (Table 2) remains similar (supplementary Table S3).

**Figure 3:**
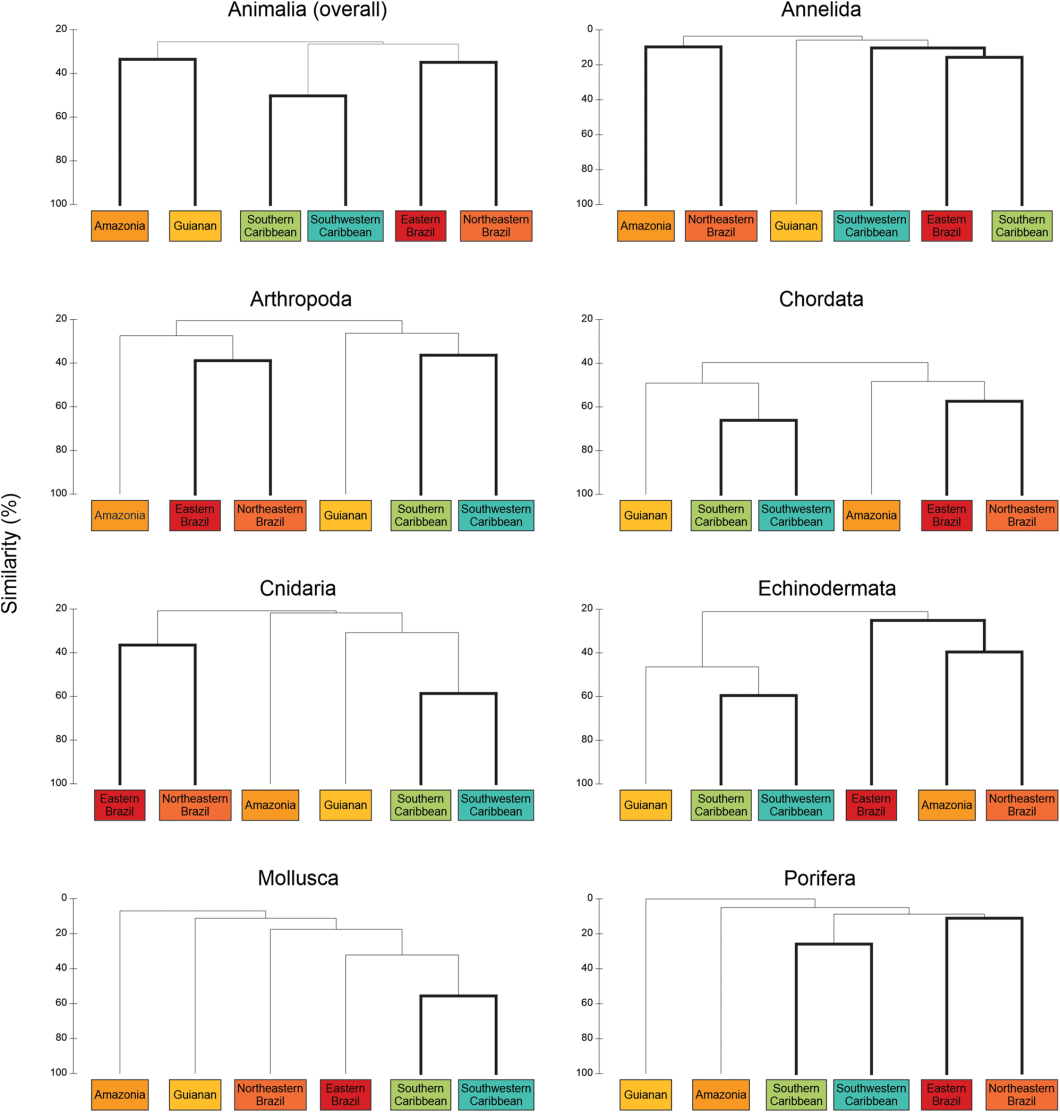
Cluster analysis dendrogram indicating patterns in species composition structure for the whole animal kingdom and the richest phyla among the six ecoregions of the Western Tropical Atlantic Ocean. Bold lines indicate significant groups in SIMPROF analysis.

Considering the whole animal kingdom (Fig. 3), we identified three biogeographic areas among the six ecoregions considered in the cluster analysis: South Caribbean (Southwestern Caribbean and Southern Caribbean), ARP (Guianan and Amazon), and Tropical Brazil (Northeastern Brazil and Eastern Brazil). Similar patterns were also observed among the richest phyla, which depicted in most cases the ecoregions of the Caribbean Sea (Southern and Southwestern) and Tropical Brazil (Northeastern and Eastern) in isolated significant branches with low similarity between each other (approximately 30% when considering the whole kingdom; Fig. 3). Exceptions occurred in Annelida and Mollusca where Eastern Brazil was more similar to Caribbean ecoregions than to Northeastern Brazil and Amazon. The two ecoregions under the influence of the ARP: Guianan and Amazon, presented different configurations in each cluster. In the whole kingdom, they were grouped together in a branch isolated from the Caribbean Sea and remaining Brazil. In Chordada, Cnidaria, Mollusca and Porifera, the two ecoregions were also isolated from others, but each in a single significant branch. Finally, in Arthropoda and Echinodermata clusters, Amazonia was grouped with the remaining Brazilian ecoregions while Guianan was isolated alone (Fig. 3).

**Figure 4:**
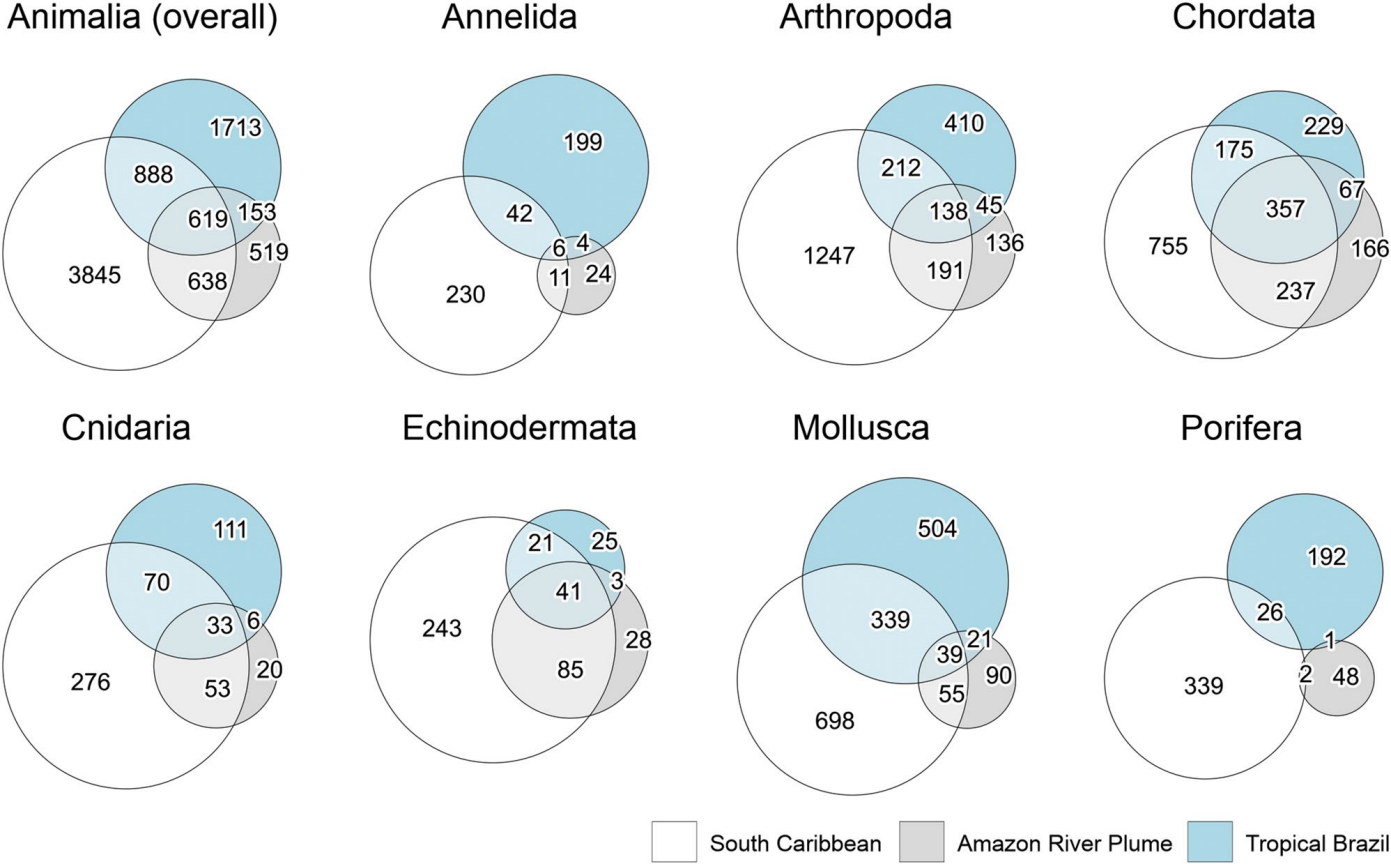
Euler diagrams indicating the number of exclusive and shared species of all the animal kingdom and the richest phyla among the three areas depicted in the cluster analysis. The size of the circles is proportional to the total number of species in each area.

Considering the three biogeographic areas defined by the cluster analyses above. Among the 5990 animal species present in South Caribbean, 64.2% were found exclusively in the area (taking in account only the ecoregions of the present work) and 21% were shared with ARP area, and 25.2% with Tropical Brazil (10.3% with both; Fig. 4, Table 3). Tropical Brazil presented 1,713 exclusive animal species i.e., 50.8% of the 3,373 present in the area. The area shared 44.7% of its animal species with South Caribbean and 22.9% with ARP (18.4% with both). ARP area presented 1,929 (26.9%) exclusive species and shared 65.16% with South Caribbean and 40% with Tropical Brazil (33.2% with both).

**Table 3:** Summary exclusive and shared animal species (%) in each area depicted in the cluster analysis (relative to the number of species observed in the area).

Phylum	Exclusive			Shared					
	SC	ARP	TBR	SC		ARP		TBR	
				ARP	TBR	SC	TBR	SC	ARP
Annelida	79.6	53.3	79.3	5.9	16.6	37.8	22.2	19.1	4
Arthropoda	69.7	26.7	50.9	18.4	19.6	64.5	35.9	43.5	22.7
Brachiopoda	66.7	0	100	33.3	0	100	0	0	0
Bryozoa	100	100	100	0	0	0	0	0	0
Chaetognatha	0	33.3	58.8	50	100	11.1	66.7	11.8	35.3
Chordata	49.5	20.1	27.7	39	34.9	71.8	51.3	64.3	51.2
Cnidaria	63.9	17.9	50.5	19.9	23.8	76.8	34.8	46.8	17.7
Echinodermata	62.3	17.8	27.8	32.3	15.9	80.3	28	68.9	48.9
Kinorhyncha	100	–	–	0	0	–	–	–	–
Mollusca	61.7	43.9	55.8	8.3	33.4	45.9	29.3	41.9	6.6
Nematoda	100	–	100	0	0	–	–	0	0
Nemertea	100	–	–	0	0	–	–	–	–
Platyhelminthes	100	–	–	0	0	–	–	–	–
Porifera	91.4	87.3	86.1	1.6	8.1	10.9	9.1	13.5	2.2
Rotifera	–	50	92.3	–	–	0	50	0	7.7
Sipuncula	78.6	66.7	66.7	7.1	14.3	33.3	0	33.3	0
Tardigrada	–	–	100	–	–	–	–	0	0
Xenacoelomorpha	100	–	–	0	0	–	–	–	–
**Total**	**64.2**	**26.9**	**50.8**	**21**	**25.2**	**65.2**	**40**	**44.7**	**22.9**

*SC* South Caribbean, *ARP* Amazon River plume, *TBR* Tropical Brazil.

Among the richest phyla, Porifera and Annelida presented the largest percentage of exclusive species in the 3 areas, ranging from 86.1 to 91.4% in the former and 53.3 to 79.6 in the latter (Fig. 4, Table 3). Meanwhile, Chordata presented the higher rates of species sharing between areas (52.1% on average), followed by Echinodermata (45.7%), Cnidaria (36.6%), Arthropoda (34.1%) and Mollusca (27.6%; Table 3).

**Figure 5:**
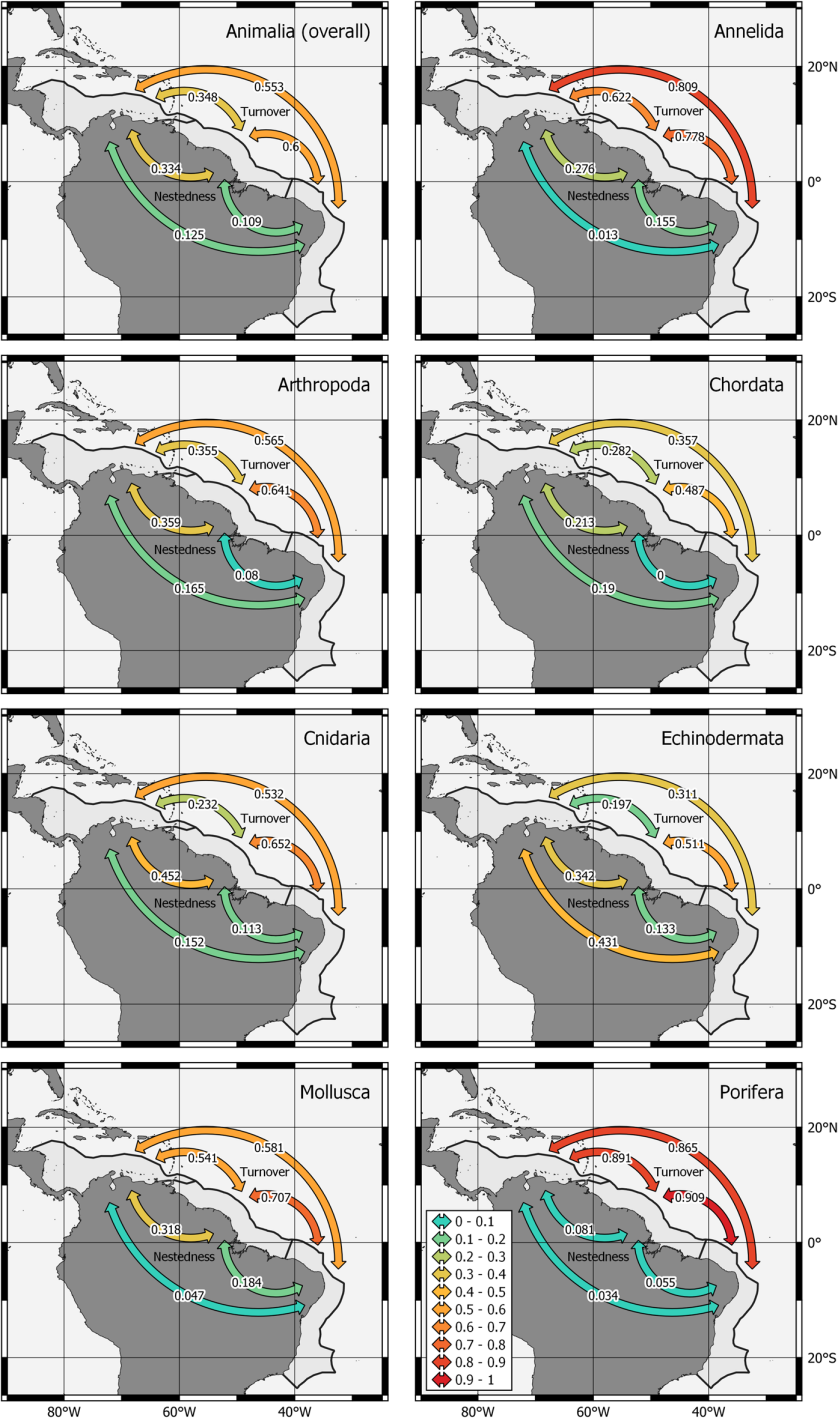
Beta diversity turnover (upper arrows) and nestedness (bottom arrows) components among the three areas depicted in the cluster analysis for the animal kingdom and the richest phyla. Maps were developed with QGIS 3.16.5 (https://www.qgis.org).

Beta diversity partitioning patterns for the whole animal kingdom and Arthropoda, Chordata, Cnidaria, and Mollusca were similar (Fig. 5). In these phyla, turnover was the main factor responsible (values at least 85% higher than nestedness) for the differences in species composition between Tropical Brazil and both ARP (ranging from 0.49 in Chordata to 0.71 in Mollusca) and South Caribbean (ranging from 0.36 in Chordata to 0.58 in Mollusca); while nestedness indexes were always below 0.19. The conjunction of high turnover and low nestedness indicate high levels of species replacement^27,28^ between Tropical Brazil and the other areas in these phyla. In contrast, both turnover and nestedness were relevant between ARP and South Caribbean in the phyla mentioned above, indicating that although part of the biodiversity was replaced (turnover ranging from 0.23 to 0.541), a higher number of species from ARP were present in South Caribbean (nestedness ranging from 0.28 to 0.45; Fig. 5). Contrasting patterns were observed in the remaining phyla. In Porifera, reflecting the high number of exclusive species, turnover was high (> 0.86) between all the areas and nestedness was virtually null. Annelida presented high turnover and low nestedness among the areas as well (except between ARP and South Caribbean; Fig. 5). Meanwhile, Echinodermata presented low turnover and high nestedness between South Caribbean with both Tropical Brazil and ARP indicating a large portion of the species from the two areas were present in South Caribbean (Fig. 5).

## Discussion

By using an extended database of 175,477 occurrences of 8,375 animal species, we broaden previous works that focused on specific phylum^3,9,17,18^ and show that the Amazon River plume (ARP) is a barrier to animal dispersal in the Western Tropical Atlantic. Before discussing the reasons and consequences of such biogeographical feature, we address potential limits intrinsic to our approach.

Any large database, including the one we used (OBIS), may present erroneous species identification and shortage of adequate data for a particular area or taxa. Since data published through OBIS must come from credible, authoritative sources and pass through a series of technical controls and reviews^29^, the first drawback is likely reduced. Among the six ecoregions considered herein, Southern and Southwestern Caribbean and Eastern Brazil have been more studied^30,31^. There, as observed in the species accumulation curves and the higher number of records in OBIS (Fig. 2, Table 1), knowledge on animal biodiversity is advanced in phyla such as Chordata, Arthropoda and Mollusca, which traditionally were extensively studied worldwide^32^. Conversely, North and Northeast Brazil are amongst the least studied regions, particularly when considering non-crustacean marine invertebrates (Fig. 2)^31,33^. Although increasing the knowledge on the biodiversity in these areas certainly would increase robustness of the present analysis, the general patterns we found were consistent in both better-studied phyla, such as Chordata and Arthropoda, and less studied ones such as Cnidaria and Echinodermata (Figs. 3, 4, 5). The situation is slowly changing as the number of studies on marine biodiversity increased in the recent years in northeast e.g.^34–36^ and north Brazil e.g.^37–39^. Thus, including their distribution records in OBIS database is essential to further improve future analyses. The Amazonian Shelf is also receiving more attention from scientific and public societies because of the presence of hard-bottom reefs, however, studies describing biological communities inhabiting this system just started to be released^14–16^.

Species richness in Caribbean ecoregions is remarkable. Both ecoregions presented at least 44% more animal species than the others. The Caribbean Sea is known to have the highest biodiversity in the Atlantic Ocean and is considered a hotspot for global conservation^30,40^. Reasons for this are still being discussed. Studies based on unique genetic sequences (haplotypes) of reef fish indicated that the Caribbean Sea is both a centre of origin, where in situ speciation occurs, and a centre of accumulation of species originated from elsewhere^5,41–43^. Intermediate levels of primary production (associated to remnants of the Amazon and Orinoco river plumes that reach the area) and natural disturbance preventing competitive exclusion may enhance in situ speciation and were previously associated to the high biodiversity of phytoplankton in the Caribbean Sea^44^. Additionally, many reef fish from the Caribbean Sea present higher frequency of basal haplotypes than other areas in the Atlantic Ocean, supporting the centre of origin hypothesis^43^. However, molecular studies evinced that South Atlantic and even Indo-Pacific haplotypes of reef fish are present in the Caribbean Sea as well, supporting the centre of accumulation hypothesis^5,41,43^. Therefore, the combination of the two hypotheses likely explains the high biodiversity observed in Caribbean ecoregions.

We show that South Caribbean and Tropical Brazilian coasts presented distinct biogeographic patterns in terms of animal communities (Fig. 3), likely isolated by the areas under the influence of the ARP. The result was cohesive in five of the seven richest phyla, which presented at least 56% dissimilarity and 27.7% of endemism between Caribbean and Tropical Brazil ecoregions (Fig. 4, Table 3). At least 10% endemism at the species level within published species inventories is an accepted practice defining biogeographic provinces^45^. Our results are thus in accordance with previous works associating the high endemism in Brazilian and Caribbean coastal habitats to the soft barrier of the ARP^3,9,17,18^, further extending the pattern to the whole animal kingdom. Species sharing among areas in the remaining 11 phyla was also low (Table 3). However, the low number of occurrences in these phyla (less than 100 records in most) hinders the possibility to infer biogeographical patterns among then.

Previous analysis indicated that the whole Caribbean Sea and Brazilian coast share 42 and 74% of their reef fish species with each other, respectively^46^. We observed similar results when considering species from the whole Chordata phylum, with the South Caribbean sharing 34% of species with Tropical Brazil, and Tropical Brazil sharing 66% of species with the South Caribbean (Fig. 4, Table 3). Considering that OBIS Chordata data include non-reef fish, and other vertebrates such as marine birds, many with large dispersal capacity, the lower percentage of shared species between the two areas reveals an even higher segregation than previously supposed.

Although the seven richest phyla showed a degree of isolation above the threshold to consider South Caribbean, ARP, and Tropical Brazil as distinct biogeographic provinces^45^, we observed particularities in the level of isolation related to the biological traits of each phylum. Soft barriers as the ARP can act as filters by restricting dispersal but at the same time allowing occasional crossings that may lead to the establishment of new populations^3^. Many biological/ecological traits are typically associated to range expansion across oceanic soft barriers in general and the ARP in particular. The position and relation with currents in the water column (i.e., nekton, plankton, or benthos), the duration of planktonic stage in meroplanktonic species and the spawning mechanism (e.g. pelagic, demersal) influence the ability of organisms to actively swim or be transported by currents across barriers^4^. Tolerance to wide range of salinity and other environmental parameters facilities survival while crossing^4^. Greater body size often means faster growth, greater competitive ability, enhanced predator avoidance and tolerance to environmental changes^4^. Multi habitat use grants more areas to be used as stepping stones to facilitate crossing and enhance establishment capacity after crossing^4^. Additionally, organism may raft with floating debris or live upon larger swimming animals crossing the barrier^4,47,48^.

Chordata was the phylum with greatest number of shared species and lowest turnover indexes. The majority (91%) of the species in the Phylum were fish (supplementary Table S1), a group with high diversity in the ecological traits presented above and mostly composed by nektonic active swimmers^4^. Thus, a higher number of species with the ability to cross the filters imposed by the ARP and/or actively use the hard bottom reefs located under it as stepping stones is expected^9^. Arthropoda, Cnidaria, and Mollusca presented similar patterns with slightly higher species turnover than Chordata. The three phyla have holoplanktonic representatives with high potential to be dispersed by currents both in coastal areas and in the open ocean, outside the brackish waters limits of the ARP^49,50^. Benthonic taxa of these three phyla typically possess planktonic larvae and the medusa stage in the case of cnidarians, which may remain weeks to months in plankton as well^47,51^. However, these meroplanktonic stages are usually more restricted to inshore waters and may require additional adaptations for survival in the low salinity surface water of the ARP or living in the deeper saline waters bellow its influence.

Interestingly, in Echinodermata, while Tropical Brazil shared 68.9% of its species with South Caribbean, South Caribbean shared only 15.9% with Tropical Brazil (Table 3). Although adult stages of the phylum are benthic with reduced mobility, pelagic larvae may persist several months in the plankton before settlement^52^. The long larval period favours its passive dispersal through currents and mesoscale meanders. In addition, the high water content of their tissues, which facilitates passive buoyancy and reduces metabolism^53^, may further enhance this capacity. In Northeast Brazil, north of 11°S, strong western boundary currents, i.e. the North Brazilian Undercurrent, the North Brazilian Current and the Guiana Current, flow parallel to coastlines northwestward in direction to Caribbean^54^. This flow likely carries larvae of Tropical Brazilian Echinodermata species across the ARP, reaching the Caribbean, while the opposite hardly occurs, what was previously suggested to occur with some invasive fish species in the Caribbean Sea that did not reach the Brazilian coast^55^.

Although more evident in Echinodermata, in all the richest phyla and when considering the whole kingdom as well, Tropical Brazil always shared relatively more species with Caribbean than the opposite (Table 3). Moreover, Tropical Brazil shared relatively more species with ARP than the South Caribbean with ARP, and ARP shared more species with the South Caribbean than with Tropical Brazil (Fig. 4, Table 3), i.e. sharing of species was always greater northwestward. This trend evinces the circulation pattern in the Western Tropical Atlantic as an important mechanism to understand the dispersal of South Atlantic animal species through the Amazon area in direction to the Caribbean Sea. Our results support the hypothesis that the Caribbean Sea is a centre of species accumulation, where currents reaching the semi-enclosed area continuously add drifting larvae of new species, which settle and accumulate there, enhancing its biodiversity^43,55^. Additionally, in the cluster analyses (Fig. 3), Amazonia ecoregion was grouped with Tropical Brazil ecoregions in Arthropoda and Echinodermata, indicating similar communities. Thus, our results indicate the northwestward circulation as an important source of holo- and meroplanktonic species for Amazonia as well.

The phylum Porifera presented the least number of shared species among the three areas, what was indicated by the highest turnover indexes (up to 0.91; Figs. 4 and 5, Table 3). Dispersal in this phylum is limited since sponges are sessile and the pelagic larval stage, when present, is very short, usually less than 2 days^56^. Thus, this phylum is likely to be more affected by the presence of the ARP. Species turnover was also particularly high in Annelida (Figs. 4, 5, Table 3). Although some Polychaeta families present holoplanktonic life cycle and high dispersal capacity^57^ they are less diversified and poorly studied^33,58^. Thus, benthic polychaete are more representative, which, as in Porifera, have generally a very short pelagic larval stage when present^57^, limiting its dispersion across the ARP.

In conclusion, by considering 8375 animal species from 175,477 OBIS records we provide the most integrative analysis on the potential of the ARP as a soft biogeographic barrier for animal dispersion in the Western Tropical Atlantic. We showed that the Caribbean Sea and Tropical Brazil continental shelves present distinct animal community structure suggesting they are isolated by the area under influence of the ARP. However, beyond species isolation due to niche difference such as low salinity intolerance, the dominant northwestward currents flowing parallel to the coast seem to play a major role in animal species dispersion among regions, enhancing the flux of larvae and other planktonic organisms with reduced mobility from Brazil to Caribbean and hindering their movement in the reverse way. The Amazon area is a stronger barrier for taxa with reduced dispersal capacity such as Porifera and Annelida, while species of pelagic taxa with active swimming may transpose it more easily.

## Methods

### Study area

We considered six ecoregions from the marine ecoregions of the World polygon data set^25^ along the Tropical Western Atlantic coast (Fig. 1): two ecoregions (Amazon and Guianan) under influence of the ARP, the two contiguous to the north in the Caribbean Sea (Southern Caribbean and Southwestern Caribbean), and to the south in Tropical Brazil (Northeastern Brazil and Eastern Brazil).

Although these regions are under the influence of western boundary current systems, distinct oceanographic characteristics and processes are observed in each of them. In both ecoregions of Tropical Brazil, narrow continental shelves (with exception of the Abrolhos bank), dominance of western boundary currents, reduced upwelling, and absence of large river discharges, result in oligotrophic waters with low primary production^19,20,59^. Coral reefs are present along the entire continental shelf.

Western boundary currents also dominate Amazon and Guianan regions, however, large freshwater discharge of the Amazon and other rivers, associated to the large continental shelf and complex circulation, with eddies and counter-currents, enhance primary production out to the open ocean^12,20,60^. Recently, a large mesophotic reef system potentially reaching 56,000 km^2^ present in the continental shelf off the Amazon River Mouth has attracted attention from the scientific community^14^. Although the biological communities inhabiting these reefs are still poorly known, the system may act as a corridor connecting The Caribbean Sea and Southwest Atlantic bellow the low salinity influence of the ARP^14,15^.

In the Caribbean Sea regions, the influence of strong western boundary currents is reduced and primary production is enhanced by meanders, eddies, upwelling, river discharge and hurricanes^21,61^. Large reef systems and the greatest biodiversity in the Atlantic ocean are observed^30,40^ .

### Data acquisition and processing

Animal species distribution data from the Tropical Atlantic Ocean between 30° N, 75° W and 32° S, 30° W were obtained from the mapper application in OBIS website in February 2021. Data was manipulated in R 1.3 software (R Core Team 2020a) to remove records below species level (i.e. with blank cell in species name or id column). To obtain a list of records (supplementary Table S1) and species presence/absence (supplementary Table S2) in each of the six ecoregions, we used the Intersect Geoprocessing Tool in QGIS, among the polygon shapefile of ecoregions^25^ and the point shapefile of spatial distribution of species records, thus, all records outside one of the ecoregions considered herein were excluded.

### Data analysis

In order to assess the current state of knowledge on animal biodiversity in each ecoregion at the OBIS database, we generated species accumulation curves from incidence data for the seven richest phyla and the remaining ones pooled using the iNEXT function from iNEXT package in R^62^.

To verify if the species distribution of the whole animal kingdom and the richest phyla were spatially structured according to the ARP, we used hierarchical cluster analyses based on Sørensen similarity matrix on the species presence/absence data^63^. Similarity profile analysis (SIMPROF) was employed to define thresholds in the cluster groups and its statistical significance (5% significance level)^63^. Cluster and SIMPROF analyses were performed in PRIMER 6 + PERMANOVA^63^.

To evaluate community compositional changes among areas depicted in the cluster analyses in the whole animal kingdom and the richest phyla, we used the beta diversity partitioning approach. In this method, beta diversity is separated in two distinct components: (i) turnover, reflecting replacement of species between areas, and (ii) nestedness, which indicates whether the area with smaller number of species resembles a strict subset of the species present in the species-richer area^27^. To illustrate these patterns, Euler diagrams were produced with the eulerr package in R^64^, beta diversity turnover and nestedness indices were calculated with beta.pair function from betapart package in R^65^.

## Supplementary Information

Supplementary Table S1.

Supplementary Table S2.

Supplementary Table S3.
